# Variation in prehospital ACS care within a single city: a bicentric observational study (MONAH-1 subgroup analysis)

**DOI:** 10.1186/s12873-026-01632-6

**Published:** 2026-06-02

**Authors:** Tobias Hofmann, Peter Baumann, Martin Sauer, Thomas Schilling, Christian Breitling, Claudia Schmidt, Frank Meyer

**Affiliations:** 1 Emergency Department with Shock Trauma Centre, Otto-von-Guericke University, Leipziger Str. 44, 39120 Magdeburg, Germany; 2 Department of Gastroenterology, Hepatology and Infectious Diseases, Otto-von-Guericke University, Magdeburg, Germany; 3 Department of General, Abdominal, Vascular and Transplant Surgery, Otto-von-Guericke University Magdeburg, University Hospital, Magdeburg, Germany; 4 Department of Anaesthesiology and Pain Therapy, Klinikum Magdeburg gGmbH, Magdeburg, Germany; 5 Department of Intensive Care and Emergency Medicine; Klinikum Magdeburg gGmbH, Magdeburg, Germany

**Keywords:** Prehospital care, MONAH-1 study, ACS, Emergency physicians

## Abstract

**Background:**

Acute coronary syndrome (ACS) is a time-critical medical emergency in which early guideline-based prehospital diagnosis and treatment are crucial for the subsequent care pathway. The aim of this study was to compare documented adherence to selected prehospital ACS process indicators between two provider structures operating within the same municipal EMS system.

**Methods:**

As part of the retrospective, bicentric observational study MONAH-1, all prehospital physician missions with typical ACS diagnoses in Magdeburg between 2014 and 2018 were analysed. This prespecified intra-urban subgroup analysis compared one EMS physician base staffed by MD1 with two EMS physician bases staffed by MD2. Because case retrieval was diagnosis-targeted from archived protocols rather than based on a prospectively maintained screening registry, a full flow diagram of all EMS missions could not be reconstructed reliably; endpoint-specific denominators are therefore reported in the text and tables. Multivariable analyses were adjusted for age and gender only and should be interpreted as partially adjusted exploratory models.

**Results:**

A total of 1,438 emergency physician interventions were evaluated (MD1: *n* = 661; MD2: *n* = 777). MD1 showed documented higher rates of 12-lead ECGs (76.9% vs. 43.5%; aOR 4.24 [95% CI 3.36–5.35]), ASA administration (91.4% vs. 70.9%; aOR 4.38 [3.19–6.00]) and heparin administration (92.6% vs. 68.0%; aOR 5.86 [4.21–8.16]). In the descriptive indication-positive subgroup with documented VAS ≥ 4, morphine was documented more often at MD1 (70.6% vs. 54.5%); the exploratory adjusted morphine model was based on missions with documented pain assessment (aOR 2.67 [2.04–3.50]). No significant differences were found for indication-based nitro-glycerine and oxygen administration. Prehospital dwell time was longer at MD1 (median 34 vs. 29 min; *p* < 0.001).

**Conclusion:**

Documented adherence to selected prehospital ACS process indicators differed between the two providers. MD1 showed higher documented rates for several process measures, but the retrospective design, heterogeneous documentation formats, limited case-mix adjustment, and the possibility of reverse causation for dwell time preclude causal inference or conclusions about patient benefit. The findings are hypothesis-generating and primarily relevant for local quality assurance and prospective validation.

**Trial registration:**

The study was registered retrospectively in the German Clinical Trials Register (DRKS00036944) on 27 August 2025.

## Introduction

Acute coronary syndrome (ACS) is one of the most time-critical medical emergencies, in which early diagnosis and guideline-based therapy are crucial for the morbidity and mortality of those affected. Even in the prehospital setting, the emergency physician can take essential measures, including structured clinical assessment, recording and interpretation of a 12-lead electrocardiogram, and early initiation of evidence-based drug therapy. Prehospital care quality thus plays a key role in the overall treatment pathway of patients with ACS [1, 2]. 

In Magdeburg, the capital of Saxony-Anhalt, three EMS physician bases are staffed 24/7 to ensure emergency care for the population. One base was staffed by Magdeburg University Hospital (MD1), whereas two EMS physician bases were staffed by Magdeburg Municipal Hospital (MD2). Against this background, ACS offers a relatively standardised tracer condition for comparing documented adherence to selected prehospital process indicators within one urban EMS environment while minimising macro-level differences in geography, dispatch framework, and hospital accessibility. The comparison therefore addresses provider structures, staffing patterns, and local routines within the same municipal system rather than hospital quality in a direct institutional sense.

The objective of this prespecified MONAH-1 subgroup analysis was to compare documented adherence to selected prehospital ACS process indicators between these two provider structures. Its distinct additional contribution beyond the previously published MONAH-1 region-based analysis is the intra-urban perspective: by focusing on bases operating within the same citywide EMS network, the present manuscript explores whether organisational and staffing differences within one municipal system are associated with variation in documented prehospital ACS management. Throughout the manuscript, the results are therefore framed as differences in documented process adherence rather than as a direct institutional ranking of the two hospitals.

## Patients and methods

### Study design and setting

This manuscript reports a prespecified Magdeburg subgroup analysis of MONAH-1, a retrospective bicentric observational study on prehospital emergency physician management of suspected acute coronary syndrome (ACS). The overall MONAH-1 design, case-identification strategy, and protocol-based extraction approach have been described previously in the published region-based comparison by Hofmann et al. [3]. In the present analysis, only cases managed at the three EMS physician bases in Magdeburg were evaluated. The distinct purpose of this subgroup analysis was to examine intra-urban variation between provider structures operating under the same municipal EMS framework.

### Guideline basis and quality indicators

Guideline adherence was assessed against the European Society of Cardiology recommendations applicable during the study period (2014–2018) for ST-elevation myocardial infarction and non-ST-elevation ACS [4–7]. From these documents, predefined process indicators were derived for statistical evaluation: acquisition of a 12-lead ECG; administration of acetylsalicylic acid (ASA) and heparin; morphine in patients with relevant pain; oxygen in hypoxaemia; and nitro-glycerine when clinically indicated. Drug selection, indications, and dosing were interpreted according to the contemporaneous guideline documents [2, 4–7]. For the present analysis, morphine was considered indication-positive only when a pain score of VAS ≥ 4 was documented. Oxygen was considered indicated when SpO₂ < 90% or when hypoxaemia/explicit respiratory compromise was documented. Nitro-glycerine was considered indicated when a compatible ACS presentation with preserved haemodynamic suitability (generally systolic blood pressure > 100 mmHg) was documented and no explicit contraindication, such as hypotension, suspected right ventricular infarction, or recent phosphodiesterase-5 inhibitor use, was recorded.

**Fig. 1:**
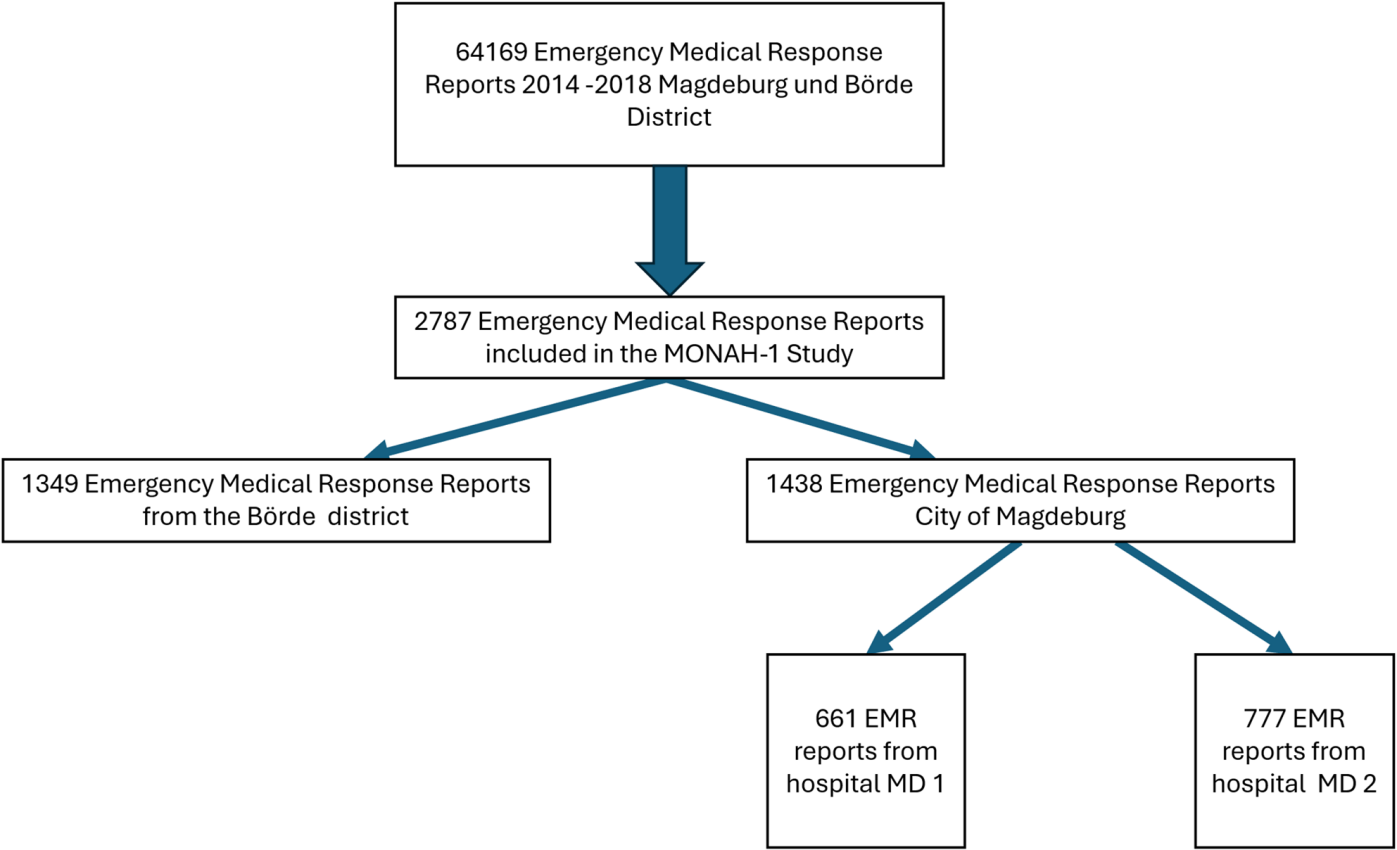
Represents an aggregated overview of the analysed cohort within the MONAH-1 dataset rather than a formal screening flow diagram of all EMS missions, as a complete reconstruction of all screened missions was not feasible due to the diagnosis-targeted archival retrieval approach. EMR: Emergency Medical Response; Hospital MD1 = Universitiy Hospital; Hospital MD2 = Magdeburg Municipal Hospital

Figure 1 represents an aggregated overview of the analysed cohort within the MONAH-1 dataset rather than a formal screening flow diagram of all EMS missions, as a complete reconstruction of all screened missions was not feasible due to the diagnosis-targeted archival retrieval approach

### Case identification

All consecutive archived prehospital emergency physician missions in Magdeburg between 2014 and 2018 were searched in a diagnosis-targeted manner for protocols documenting ACS, STEMI, NSTEMI, unstable angina, myocardial infarction, or acute myocardial infarction. Missions documented by the rescue helicopter Christoph 36 were excluded because they do not reflect routine ground-based emergency physician coverage and because the corresponding records were not available for evaluation for data-protection reasons. Because archival case retrieval was diagnosis-targeted rather than based on a prospectively logged all-mission screening register, the total number of initially screened EMS missions and detailed cause-specific exclusions beyond helicopter missions could not be reconstructed reliably for a formal flow chart.

### Setting

During the study period, one Magdeburg EMS physician base was staffed by Magdeburg University Hospital (MD1), whereas two bases were staffed by Magdeburg Municipal Hospital (MD2). Thus, the comparison reflects two provider structures operating within the same urban emergency medical service system. Relevant structural differences between both provider structures included the number of staffed EMS physician bases, the specialty composition of the physicians involved, and potentially differing local training, supervision, and documentation routines.

### Data acquisition and handling of missing data

Emergency physician protocols from the study period were reviewed in paper or electronic form, depending on the respective base and year of documentation. Both structured check boxes and free-text entries were used to identify diagnoses and prehospital measures. Tactical and demographic variables were analysed only when documented. For treatment-related variables, absence of documentation was classified as “not documented”/not documented for the purpose of process evaluation. For example, a 12-lead ECG was counted only when ECG acquisition or corresponding findings were explicitly recorded in the structured fields or free text. This rule was defined a priori and is consistent with the medico-legal principle that undocumented measures cannot be assumed to have been documented [8]. Because documentation formats differed between sites and years, differential misclassification is a central methodological concern. Site-specific evaluable denominators are therefore reported for endpoint analyses (Table 3), and model-specific denominators are reported in Table 4. Pain intensity for the descriptive morphine indicator was abstracted from the documented VAS field or an equivalent free-text entry. Missions without documented pain assessment were excluded from the descriptive indication-positive morphine denominator rather than classified as no pain or no indication.

### Statistical analysis

Data were entered into Microsoft Excel and analysed with IBM SPSS Statistics version 29. Categorical variables are reported as absolute and relative frequencies and were compared using chi-square tests or Fisher’s exact tests as appropriate. Continuous variables are presented as medians with interquartile ranges and were compared using the Mann-Whitney U test. To examine whether the EMS physician location was independently associated with selected prehospital measures, multivariable logistic regression models were calculated with adjustment for age and sex. Results are reported as adjusted odds ratios (aORs) with 95% confidence intervals (CIs). All statistical tests were two-sided, and *p* < 0.05 was considered statistically significant. Because the archival dataset did not permit robust adjustment for additional case-mix variables or clustered modelling at physician/base level, all multivariable analyses should be understood as partially adjusted exploratory models that treat missions as independent observations. For transparency, Table 4 reports the endpoint-specific number of missions included in each model; lower model N reflects endpoint-specific eligibility, documentation completeness, and the availability of age/sex covariates. For morphine, the descriptive comparison in Table 3 was restricted to VAS ≥ 4, whereas the exploratory adjusted model used the broader subgroup with documented pain assessment.

## Results

A total of 1,438 emergency physician protocols were used for statistical analysis in this article. Of these, 661 patients were treated by emergency physicians at the University Hospital provider structure (MD1) and 777 patients were treated by emergency physicians at the Magdeburg Municipal Hospital provider structure (MD2). Tables 1 and 2 show the patient characteristics (gender distribution, age) and emergency physician characteristics (specialisation and qualifications of the emergency physicians deployed). Because case retrieval was diagnosis-targeted from archived records, a formal screening flow chart of all EMS missions could not be reconstructed reliably. Endpoint-specific analytic denominators are therefore given in Tables 3 and 4. Full-cohort denominators were available for 12-lead ECG, ASA, and heparin; eligible denominators were 1,323 missions for nitro-glycerine, 131 for oxygen, 518 for the descriptive morphine analysis with documented VAS ≥ 4, and 922 for the exploratory adjusted morphine model with documented pain assessment. The difference between the full cohort and the regression-model denominator of 1,415 for ECG/ASA/heparin reflects missing age and/or sex data in 23 missions.

**Table 1:** Patient characteristics

Variable	MD1 (*n* = 661)	MD2 (*n* = 777)	*p* value
Age, years (median [IQR])	71 [60–79]	72 [60–80]	0.334
Gender, n (%)			0.004
Male	375 (56.7%)	483 (62.2%)	
Female	286 (43.3%)	286 (36.8%)	
Unknown	0 (0.0%)	8 (1.0%)	

Continuous variables were compared using the Mann–Whitney U test. Categorical variables were compared using the chi-square test. A two-sided p value < 0.05 was considered statistically significant

**Table 2:** Emergency physician characteristics

Variable	MD1 (*n* = 661)	MD2 (*n* = 777)	*p* value
Speciality, n (%)			0.001
GM	0	2 (0.3%)	
AN	661 (100.0%)	565 (72.7%)	
SU	0 (0.0%)	128 (16.5%)	
IM	0 (0.0%)	68 (8.8%)	
UK	0 (0.0%)	14 (1.8%)	
Qualification, n (%)			0.001
R	369 (55.8%)	347 (44.7%)	
S	292 (44.2%)	416 (53.5%)	
UK	0 (0.0%)	14 (1.8%)	

Categorical variables were compared using the chi-square test. A two-sided p value < 0.05 was considered significant. GM: general medicine; AN: anaesthesia; SU: surgery; IM: internal medicine; UK: unknown; R: resident; S: specialist

Further analyses revealed differences in documented prehospital process measures between the two provider structures under review.

The subgroup analysis showed that emergency physicians at MD1 documented 12-lead ECG acquisition more often than emergency physicians at MD2 (76.9% vs. 43.5%; OR 4.31 [95% CI 3.43–5.43]; *p* < 0.001). The administration of ASA (91.4% vs. 70.9%; OR 4.35 [3.18–5.94]; *p* < 0.001) and heparin (92.6% vs. 68.0%; OR 5.89 [4.24–8.17]; *p* < 0.001) was also more common among emergency physicians at MD1. In patients with documented pain intensity of VAS ≥ 4, morphine was documented by emergency physicians at MD1 in 70.6% of cases, compared with 54.5% by emergency physicians at MD2 (OR 2.01 [1.33–3.05]; *p* < 0.001). In contrast, there were no differences between the two provider structures in the indication-appropriate administration of nitro-glycerine (50.6% vs. 54.8%; OR 0.85 [0.68–1.05]; *p* = 0.136) or oxygen (72.7% vs. 78.5%; OR 0.73 [0.33–1.63]; *p* = 0.445). The results are given in Table 3. Expressed as absolute differences, the documented between-site gaps were + 33.4% points for 12-lead ECG, + 20.5 for ASA, + 24.6 for heparin, and + 16.1 for morphine when pain intensity was documented; the absolute differences for nitro-glycerine and oxygen were − 4.2 and − 5.8% points, respectively. Table 4 should be interpreted separately for morphine, because its exploratory adjusted model was fit in the broader subgroup with documented pain assessment (*N* = 922) rather than only in the VAS ≥ 4 subgroup.

**Table 3:** Measures taken (subgroup analysis) in MD1 and MD2

Variable	MD1	MD2	OR (95% CI)	*p*-value
12-lead ECG	508/661 (76.9%)	338/777 (43.5%)	4.31 (3.43–5.43)	< 0.001
Morphine administration (documented VAS ≥ 4)	279/395 (70.6%)	67/123 (54.5%)	2.01 (1.33–3.05)	< 0.001
Nitro-glycerine administration when indicated	313/618 (50.6%)	386/705 (54.8%)	0.85 (0.68–1.05)	0.136
ASA administration	604/661 (91.4%)	551/777 (70.9%)	4.35 (3.18–5.94)	< 0.001
Heparin administration	612/661 (92.6%)	528/777 (68.0%)	5.89 (4.24–8.17)	< 0.001
Oxygen administration when indicated	48/66 (72.7%)	51/65 (78.5%)	0.73 (0.33–1.63)	0.445

Notes: Values as n/N (%). Site-specific evaluable denominators are shown explicitly because endpoint eligibility and documentation completeness differed across indicators. Morphine denotes the descriptive indication-positive subgroup with documented VAS ≥ 4. Odds ratio (OR) for MD1 compared with MD2. p-values from chi-square test; for small expected frequencies, Fisher’s exact test. 95% confidence intervals of OR according to Woolf; for zero cells, Haldane-Anscombe correction

The multivariate adjusted analysis (adjustment for age and gender) showed similar directionality for 12-lead ECG, ASA, and heparin and an exploratory association for morphine administration within the pain-documented subgroup. MD1 remained associated with more frequent documentation of 12-lead ECGs (aOR 4.24 [3.36–5.35]), ASA administration (aOR 4.38 [3.19–6.00]), heparin administration (aOR 5.86 [4.21–8.16]), and morphine administration among missions with documented pain assessment (aOR 2.67 [2.04–3.50]; *p* < 0.001 in each case). There were no differences even after adjustment for O₂ administration in cases of hypoxaemia (aOR 0.76 [0.33–1.74]; *p* = 0.516) and nitroglycerine administration when indicated (aOR 0.84 [0.67–1.04]; *p* = 0.110). These results are shown in Table 4. Because covariate availability was limited, these adjusted models should be interpreted as partially adjusted exploratory models rather than fully case-mix-adjusted estimates. In addition, the reported confidence intervals may be overly narrow because clustering within EMS bases and individual physicians could not be modelled. Because missions are likely clustered within EMS bases and individual physicians, the absence of hierarchical modelling may result in underestimated variance and overly narrow confidence intervals.

**Table 4:** Multivariate analysis MD1 vs. MD2 – prehospital treatment (adjusted for age and gender)

Measure	*N* (in model)	aOR (95% CI)	*p*-value
12-lead ECG	1415	4.24 (3.36–5.35)	< 0.001
ASA administration	1415	4.38 (3.19–6.00)	< 0.001
Heparin administration	1415	5.86 (4.21–8.16)	< 0.001
Morphine administration among missions with documented pain assessment	922	2.67 (2.04–3.50)	< 0.001
O₂ administration when indicated	128	0.76 (0.33–1.74)	0.51
Nitro-glycerine administration when indicated	1304	0.84 (0.67–1.04)	0.11

Abbreviations: aOR = adjusted odds ratio; CI = confidence interval. Model-specific N reflects endpoint-specific eligibility, documentation completeness, and availability of age/sex covariates. The morphine model denominator differs from Table 3 because it was based on the broader pain-documented subgroup

The PHVZ (prehospital dwell time), defined as the time from arrival at the patient to handover at the hospital, was analysed as a potentially relevant operational characteristic. It differed significantly between the two provider structures. Emergency physicians at MD1 had a longer PHVZ than emergency physicians at MD2 (median 34 [IQR 29–39] vs. 29 [23–34] minutes; *p* < 0.001). This variable should be interpreted as an associated operational feature rather than as an explanatory or causal variable in the current retrospective dataset. Any interpretation suggesting that longer dwell time reflects improved care would be speculative and cannot be supported by the present data.

### Summary of results

A total of 1,438 emergency physician interventions were analysed (MD1: *n* = 661; MD2: *n* = 777). In the guideline-compliant subgroup analysis, documented prehospital process measures by emergency physicians at MD1 hospital showed a significantly higher rate of 12-lead ECGs documented (76.9% vs. 43.5%; OR 4.31 [95% CI 3.43–5.43]; *p* < 0.001) and higher documented rates of ASA (91.4% vs. 70.9%; OR 4.35 [3.18–5.94]; *p* < 0.001) and heparin (92.6% vs. 68.0%; OR 5.89 [4.24–8.17]; *p* < 0.001).

In patients with VAS ≥ 4, morphine was documented more frequently by emergency physicians at Clinic MD1 than by emergency physicians at Clinic MD2 (70.6% vs. 54.5%; OR 2.01 [1.33–3.05]; *p* < 0.001). In contrast, there were no significant differences in documented prehospital process measures between the emergency physicians at clinics MD1 and MD2 when nitro-glycerine and oxygen were documented as indicated.

The multivariate adjusted analysis (for age and gender) showed similar associations after adjustment for age and sex. In addition, the prehospital dwell time for emergency physicians at Clinic MD1 was significantly longer than for emergency physicians at Clinic MD2 (median 34 [IQR 29–39] vs. 29 [23–34] minutes; *p* < 0.001).

## Discussion

This intra-urban subgroup analysis of MONAH-1 identified substantial between-site variation in documented adherence to selected prehospital ACS process indicators between two provider structures operating within the same municipal EMS system. Compared with MD2, emergency physicians at MD1 more frequently documented a 12-lead ECG and more often documented ASA, heparin, and morphine-related process measures. By contrast, no relevant differences were observed for indication-based nitroglycerine or oxygen administration. Because adjustment was limited and documentation was heterogeneous, these findings should be interpreted as differences in documented process adherence rather than as direct evidence of superior hospital or provider performance. Documentation bias remains a central methodological concern, because some between-site differences may reflect variation in recording completeness or documentation culture rather than true differences in delivered care. The magnitude of the observed effect sizes should be interpreted with particular caution, as unmeasured confounding factors such as case severity, dispatch prioritization, and operational context may substantially influence these associations. Given that undocumented measures were classified as not documented, even moderate differences in documentation practices between sites may lead to substantial apparent differences in process adherence, potentially limiting internal validity.

The observed differences are clinically relevant at the process level because early ECG documentation and indication-based administration of antithrombotic and analgesic therapy can influence downstream triage, catheterisation-laboratory activation, and symptom control. However, the absence of patient-centred outcomes means that the present data cannot show whether the documented between-site differences translated into faster reperfusion, fewer complications, or lower mortality. Process quality in this dataset should therefore not be equated with proven clinical superiority. Higher rates of documented interventions should not be interpreted as inherently superior quality of care, but must be considered in the context of clinical indication and the potential for overtreatment.

The observed differences in morphine administration between the two provider structures should be interpreted in the context of the ongoing debate regarding the safety and clinical impact of opioid use in acute coronary syndrome. Several meta-analyses have raised concerns that morphine may be associated with adverse outcomes, including potential interference with antiplatelet drug absorption and a possible increase in mortality or reinfarction rates, although the evidence remains heterogeneous and partly confounded [9, 10]. At the same time, other analyses suggest that morphine can be used safely when clinically indicated, particularly for adequate pain control, highlighting the persistent uncertainty and the need for individualised decision-making in the prehospital setting. Against this background, the higher documented morphine use at MD1 cannot be interpreted as either clearly beneficial or harmful, but rather reflects variation in clinical practice within an area of ongoing scientific debate.

Beyond pharmacological considerations, differences in analgesic use may also be influenced by provider-related factors. Previous work in the German EMS system has demonstrated that analgesic administration patterns vary according to physician specialty, training background, and clinical experience [11]. This aligns with the present findings, where MD1 was staffed exclusively by anaesthesiologists, while MD2 involved a broader mix of specialties. Differences in training among prehospital emergency physicians in Germany have been identified as a relevant source of variability in clinical decision-making and guideline adherence [12].

In addition, alternative organisational models such as telemedically supported paramedic systems have been shown to achieve guideline adherence comparable to, or in some cases exceeding, conventional on-scene physician-based care [13]. Longitudinal data further suggest that structured system-level interventions, including telemedicine integration and standardised treatment algorithms, may improve adherence to evidence-based ACS management over time [14]. These findings support the interpretation that organisational structure and system design—rather than individual provider performance alone—play a key role in shaping prehospital care quality.

Finally, retrospective real-world analyses of prehospital ACS care have consistently demonstrated substantial variability in guideline adherence, even within comparable healthcare systems [15]. The present intra-urban findings are therefore consistent with the broader literature and reinforce the interpretation that the observed differences likely reflect a complex interplay of pharmacological uncertainty, provider training, and system-level organisational factors rather than isolated performance differences.

Importantly, the clinical relevance of these process indicators is not confined to the historical guideline period covered by this dataset. Current ACS guideline recommendations continue to emphasise rapid ECG-based triage, early indication-based antithrombotic treatment, and a restrictive use of supplemental oxygen limited to patients with hypoxaemia. Against this background, the between-site variation observed here concerns process domains that remain central to contemporary ACS care pathways, which further strengthens the rationale and present-day relevance of the study [16].

In addition, recent ACS literature highlights the relevance of early risk stratification beyond the initial diagnostic categorisation. In patients with non-ST-segment elevation ACS, Demirtas Inci et al. showed that combining the QTc interval with the GRACE risk score improved prediction of early mortality, supporting the broader concept that early ECG-derived information may contribute not only to diagnosis but also to prognostic assessment [17]. Although QTc and GRACE data were not available in the present prehospital dataset, this finding further underlines the potential importance of obtaining interpretable ECG information as early as possible in the ACS pathway.

The longer prehospital dwell time observed at MD1 should remain hypothesis-generating rather than explanatory. It may have been associated with more opportunity for ECG acquisition, analgesia titration, and antithrombotic initiation, but it may equally reflect reverse causation, greater case complexity, differing dispatch or transport characteristics, local routines, or more complete documentation. The present analysis cannot disentangle these possibilities. Any directional or causal interpretation would therefore be inappropriate.

Documentation bias deserves particular emphasis. Because protocols were reviewed in paper or electronic form depending on site and year, and because undocumented care was classified as not documented for process evaluation, systematic differences in recording culture or form design between MD1 and MD2 may have produced differential misclassification. Some of the observed between-site gaps may therefore reflect documentation completeness rather than true differences in delivered care.

Team composition is another plausible explanatory factor. During the study period, MD1 was staffed exclusively by anaesthesiology physicians, whereas MD2 involved physicians from several specialties. This heterogeneity may have influenced routines, algorithm familiarity, and documentation practices; however, the present dataset does not allow a robust mission-level subgroup analysis that could test this hypothesis directly. It should therefore be regarded as a plausible but unproven explanatory structure.

From a quality-assurance perspective, the findings are best understood as evidence of remediable process variation within one urban EMS system. Repeated audit cycles, standardised documentation rules, harmonised protocol templates, and focused refresher training could reduce unwarranted between-site variation while simultaneously improving data quality for future evaluations.

The distinct additional contribution of this manuscript beyond the previously published MONAH-1 region-based comparison is the intra-urban perspective: by comparing provider structures within the same citywide EMS environment, the analysis reduces macro-level urban-rural differences and highlights how local organisational routines, staffing patterns, and documentation practices may shape documented ACS process adherence even within one municipal system. Overall, the present results should be interpreted narrowly as hypothesis-generating differences in documented adherence to selected prehospital ACS process indicators.

Prospective validation with standardised electronic documentation, richer case-mix variables, clustered modelling, and linkage to patient-centred outcomes is needed before stronger inferences about provider performance or clinical benefit can be made.

## Methodological limitations

This study has several limitations. First, its retrospective design relies on routine documentation. Because the analysis was based on protocol entries rather than direct observation, documentation bias is not a peripheral issue but a central methodological concern. The use of both handwritten and electronic records across sites and years creates a substantial risk of differential misclassification, and some observed between-site differences may reflect recording completeness or documentation culture rather than true care differences.

Second, the study evaluated documented adherence to selected prehospital process indicators rather than patient-centred outcomes. Reperfusion delay, downstream treatment, complications, and mortality were not assessed, so the clinical consequence of the observed documentation-based process differences remains unknown.

Third, multivariable adjustment was limited to age and sex. Important potential confounders such as ACS subtype and clinical severity, pain intensity, haemodynamic instability, transport distance, dispatch priority, time of day/workload, receiving hospital, mission-level team composition, comorbidity burden, and formal risk markers were not available in a sufficiently complete form for robust adjustment. The models should therefore be regarded as partially adjusted exploratory analyses rather than definitive case-mix-adjusted comparisons. The magnitude of the observed effect sizes should be interpreted with particular caution, as unmeasured confounding factors such as case severity, dispatch prioritization, and operational context may substantially influence these associations.

Fourth, missions were analysed as independent observations although clustering within EMS bases and probably within individual physicians is likely. Because physician-level identifiers suitable for hierarchical modelling were not available, the reported confidence intervals may overstate precision. In addition, the study period from 2014 to 2018 was analysed as a single interval, so potential temporal trends in documentation or practice could not be examined.

Finally, data retrieval was logistically demanding because archived paper records had to be reviewed under restricted access conditions. These constraints also limited the reconstructability of a full screening flow diagram and the complete quantification of site-specific missingness for every candidate variable.

Despite these limitations, the study provides a real-world, intra-urban quality-assurance assessment of documented prehospital ACS process adherence and identifies concrete targets for prospective implementation work.

## Summary and outlook

Acute coronary syndrome (ACS) is a time-critical emergency in which early prehospital diagnosis and treatment are important for subsequent care pathways. This prespecified MONAH-1 subgroup analysis compared documented adherence to selected prehospital ACS process indicators between two provider structures within the same municipal EMS system in Magdeburg. Among 1,438 included missions, MD1 showed higher documented rates of 12-lead ECG acquisition, ASA administration, and heparin administration; morphine-related findings differed between the descriptive VAS ≥ 4 subgroup and the broader exploratory pain-documented model. No significant between-site differences were observed for indication-based nitroglycerine or oxygen administration. Because the study relied on routine documentation, used only limited case-mix adjustment, and did not assess patient-centred outcomes, the results should be interpreted as hypothesis-generating differences in documented process adherence rather than proof of superior clinical care. Prospective studies with standardised documentation, broader adjustment, and outcome linkage are needed to validate these findings.

## Data Availability

The datasets generated and/or analysed during the current study are not publicly available because they contain clinical routine data subject to data-protection and patient-privacy restrictions, but de-identified data may be available from the corresponding author on reasonable request and subject to institutional and legal approval.
